# Short-term mortality and readmission rate prediction by the sequential organ failure assessment score in acute decompensated heart failure

**DOI:** 10.1097/MS9.0000000000002852

**Published:** 2024-12-27

**Authors:** Van Hoang, Dong Tran Van, Hoa Tran Thanh, Dan Nguyen Van, Thuc Nguyen Van, Nga Vu Quynh, Giang Tran Tra

**Affiliations:** Hanoi Heart Hospital, Hoan Kiem, Hanoi, Vietnam

**Keywords:** acute decompensated heart failure, short-term mortality, SOFA score

## Abstract

**Background:**

Data on the use of sequential organ failure assessment (SOFA) in patients with cardiovascular disease are increasing. Several studies demonstrated that the SOFA score can identify short-term mortality in patients with acute decompensated heart failure (ADHF). This study was conducted to determine the prognostic value of the admission SOFA score in patients hospitalized for ADHF and to assess its ability in predicting the 30-day readmission rate.

**Materials and methods:**

This study was conducted from July 2022 to August 2023 at our institution. A total of 216 patients were included in the study, and the SOFA score was calculated for all patients.

**Results:**

The average SOFA score is 3.14 ± 2.67, of which SOFA scores of ≤1, 2–3, and ≥4 account for 34.3%, 27.8%, and 37.9% of the total score, respectively. During the 30-day follow-up period, there were 22 cases of death from all causes (10.2%), of which in-hospital mortality accounted for 9.7%. Patients with all-cause mortality had higher SOFA scores than the survivors (7.32 ± 2.93 and 2.66 ± 2.12, respectively). The area under the receiver operating characteristic curve (AUC) for the SOFA score is 0.897 [95% confidence interval (CI) = 0.830–0.964] for 30-day mortality. The SOFA score was also compared with other validated risk scores, namely, the Get With the Guidelines-Heart Failure (GWTG-HF) risk score and the Acute Decompensated Heart Failure National Registry (ADHERE) score. In this study cohort, the SOFA score demonstrates superior predictive accuracy to the GWTG-HF and ADHERE scores [the AUCs when predicting 30-day mortality are 0.769 (95% CI = 0.676–0.862) and 0.789 (95% CI = 0.705–0.873), respectively].

**Conclusion:**

The SOFA score might be used to screen high-risk patients for optimization strategy in the treatment of ADHF.

## Introduction

The sequential organ failure assessment (SOFA) score was developed and widely used as a tool to quantify the risk of in-hospital mortality in patients with sepsis in intensive care units (ICUs)[1]. The SOFA score includes a four-point assessment of the dysfunction of six organ systems (nervous, respiratory, cardiovascular, renal, hepatic, and coagulatory systems), and a SOFA score ≥2 is associated with an increased risk of adverse outcomes[2]. Although the 2021 Surviving Sepsis Campaign guidelines advise against using qSOFA and SOFA as standalone tools for sepsis screening[3], their effectiveness in other clinical scenarios, such as forecasting 30-day readmission rates and mortality in patients with acute decompensated heart failure (ADHF), has yet to be investigated. Recent studies have demonstrated the ability of the SOFA score in discriminating short-term mortality and even predicting long-term mortality in unselected patients with cardiovascular disease admitted to ICUs[2]. Compared with other prediction score systems, SOFA has the advantages of simplicity and clinical availability with electronic devices. Owing to these aforementioned results and the similarities in pathophysiological mechanisms in ADHF and septic shock, several authors have studied the prognostic ability of the SOFA score in patients hospitalized with acute heart failure^[4,5]^. However, the study populations included only limited patients with severely reduced left ventricular ejection function (LVEF) (i.e., LVEF ≤ 30%), or the majority of patients (31.2%) had missing echocardiography data[4]. Therefore, the prognostic ability of the SOFA score in patients with ADHF might be underestimated.

This study was conducted to determine the prognostic value of the admission SOFA score in patients hospitalized for ADHF and to assess its ability to predict 30-day readmission rate.

## Methods

### Patient selection

This was a single-center, prospective study conducted from July 2022 to August 2023 at our institution. All patients over 18 years old who were hospitalized for acute heart failure were included (either new-onset acute heart failure or ADHF). Patients with septic shock or pulmonary infarction were excluded.

Previous investigations have indicated that the mortality rate among patients admitted to ICUs, particularly those hospitalized with acute heart failure, approximates 10% ($\hat{p}$)[2]. Given the characteristics of a single-center, short-term study design, the sample size was determined using a margin of error of 4% (m) and a statistical significance level of 0.05 (α), resulting in the following:

$$N=\frac{{z^{2}}_{1-\alpha /2}\times \hat{p}\times \left(1-\hat{p}\right)}{m^{2}}=139.$$

### Data collection

Informed consent was obtained from all patients at the time of admission. Furthermore, at the time of admission, medical history was recorded, and clinical examination was performed. Echocardiography was performed for each patient within 60 min of admission, and LVEF was assessed in detail. Laboratory results were collected at admission and during the hospital stay.

### Risk scores

Similar to a previously published study[1], the SOFA subscores in the current study were determined separately on the basis of the severity of system impairment (neurologic, cardiovascular, renal, respiratory, coagulatory, and hepatic systems). Each system was assigned a score that ranged from 0 to 4. The overall SOFA score was generated by combining the subscores.

The Acute Decompensated Heart Failure National Registry (ADHERE) scores of patients were determined using the classification tree method via their admission systolic blood pressure (SBP), blood urea nitrogen (BUN), and creatinine levels. Patients were categorized into low, intermediate 3, intermediate 2, intermediate 1, and high-risk groups on the basis of the increasing severity of in-hospital mortality[6].

Get With the Guidelines-Heart Failure (GWTG-HF) risk score[7] was created using data from the American Heart Association GWTG-HF program and is used as a prognostic tool for in-hospital mortality in patients with acute heart failure. The risk score was assessed using the following variables: age, SBP, heart rate, BUN, sodium, race (such as African), and a diagnosis of chronic obstructive pulmonary disease.

### Heart failure management

All patients were managed with optimal tolerated medical therapy, as recommended by the European Society of Cardiology Guidelines[8].

### Statistical analysis

Mean standard deviation was used to summarize continuously distributed and normally distributed variables. Categorical variables are displayed as proportions and frequencies.

Student’s *t*-test was used to compare the continuous variables between groups, whereas analysis of variance (ANOVA) test was used to compare the means among three or more groups. Analysis of variance and the χ^2^ test were utilized when comparing continuous and categorical variables between more than two groups.

Time to death was represented using Kaplan–Meier curves. The area under the curve (AUC) of the receiver operating characteristic (ROC) curve was used to determine the predictive value for the in-hospital and 30-day mortality of the SOFA score, and the log-rank test was used for between-group comparisons.

For all analyses, *P* < 0.05 was considered statistically significant. SPSS 24.0 and STATA 14.0 were used for statistical analysis.

This research was conducted in line with the Strengthening The Reporting Of Cohort Study in Surgery (STROCSS) guidelines criteria[9].

## Results

### Baseline characteristics

From July 2022 to August 2023, 216 patients who met the inclusion criteria were continuously selected for the study (Table 1). The average age of the patients is 64.46 ± 13.72, and the majority of the patients were male (61.1%). The average LVEF is 37.69 ± 14.23%, of which LVEF < 20% accounts for 10.6% and LVEF from 20 to 30 accounts for 29.6%. The average blood creatinine concentration is 131 ± 100 mmol/dL, and renal failure was mainly observed in the group with high SOFA score ≥ 2. The average blood lactate concentration is 3.22 ± 3.32, with a significant difference between the lactate concentration in the group with SOFA score > 5 (*P* < 0.001).

**Table 1:** Distribution of demographic and clinical characteristics according to SOFA score on admission

	All 216 (100%)	SOFA score				*P* value
		Category 1 (0–1 points)	Category 2 (2–3 points)	Category 3 (4–5 points)	Category 4 (>5 points)	
		74 (34.3%)	60 (27.8%)	41 (19.0%)	41 (19.0%)	
Age	69.46 ± 13.72	71.23 ± 12.59	70.10 ± 14.94	68.15 ± 12.70	66.63 ± 14.69	0.325
Female	84 (38.9)	35 (41.7)	18 (21.4)	15 (17.9)	16 (19.0)	0.232
Male	132 (61.1)	39 (29.5)	42 (31.8)	26 (19.7)	25 (18.9)	
Hypertension	118 (54.4)	44 (20.4)	26 (12.0)	25 (11.6)	23 (10.6)	0.217
COPD	11 (5.1)	2 (0.9)	6 (2.8)	2 (0.9)	1 (0.5)	0.215
CKD	34 (15.7)	1 (0.5)	15 (6.9)	11 (5.1)	7 (3.2)	*0.001*
Diabetes	51 (23.5)	17 (7.9)	14 (6.5)	10 (4.6)	10 (4.6)	0.997
LVEF ≤ 20%	23 (10.6)	9 (4.2)	5 (2.3)	4 (1.9)	5 (2.3)	0.646
20% < LVEF ≤ 30%	64 (29.6)	24 (11.1)	19 (8.8)	10 (4.6)	11 (5.1)	
30% < LVEF ≤ 40%	42 (19.4)	9 (4.2)	11 (5.1)	10 (4.6)	12 (5.6)	
LVEF > 40%	87 (40.3)	32 (14.8)	25 (11.6)	17 (7.9)	13 (6.0)	
Creatinine (µmol/L)	131.09 ± 100.35	89.89 ± 37.03	124.83 ± 53.00	179.93 ± 165.64	166.65 ± 116.07	*0.001*
AST (U/L)	110.35 ± 249.64	92.45 ± 193.58	61.38 ± 58.31	112.38 ± 119.17	211.87 ± 481.67	*0.023*
ALT (U/L)	68.11 ± 151.66	53.79 ± 113.26	32.79 ± 23.55	71.47 ± 100.03	141.94 ± 285.12	*0.003*
NT proBNP (pmol/L)	10 521.05 ± 11 446.25	7929.68 ± 10 282.79	11 056.23 ± 10 370.64	12 374.24 ± 12 013.16	12 240.04 ± 13 617.57	*0.032*
pH (arterial blood gas)	7.39 ± 0.21	7.37 ± 0.32	7.41 ± 0.07	7.41 ± 0.08	7.38 ± 0.15	*0.030*
Lactate (mmol/L)	3.22 ± 3.32	2.56 ± 1.92	1.89 ± 0.87	3.24 ± 2.55	6.37 ± 5.67	*0.001*
CRP (mg/dL)	21.83 ± 33.40	9.03 ± 13.98	25.62 ± 36.88	31.02 ± 40.9	21.83 ± 33.4	*< 0.001*

ALT, alanine transaminase; AST, aspartate aminotransaminase; CKD, chronic kidney disease; COPD, chronic obstructive pulmonary disease; CRP, C-reactive protein; LVEF, left ventricular ejection fraction; NT-proBNP, N-terminal prohormone of brain natriuretic peptide; SOFA, sequential organ failure assessment.

Values are reported as mean ± standard deviation or number of patients (%) unless otherwise noted.

Italic values represent *p*-value less than 0.05 is statistically significant.

**Figure 1.**
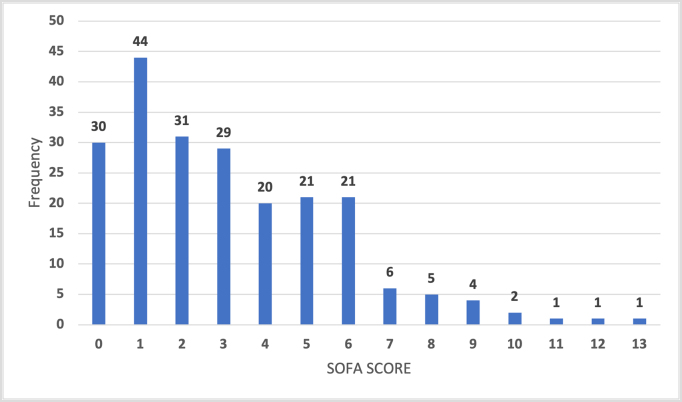
SOFA score frequency distribution. SOFA, sequential organ failure assessment.

The average SOFA score is 3.14 ± 2.67, of which SOFA scores of ≤1, 2–3, and ≥4 account for 34.3%, 27.8%, and 37.9% of the total, respectively (Fig. 1). During the 30-day follow-up period, there were 22 cases of death from all causes (10.2% of cases), of which in-hospital mortality accounted for 9.7%. Patients with all-cause mortality had higher SOFA scores than the survivors (7.32 ± 2.93 and 2.66 ± 2.12, respectively).

The AUC of the ROC curves for SOFA score is 0.897 [95% confidence interval (CI) = 0.830–0.964] for 30-day mortality. The SOFA score was also compared with the GWTG-HF and ADHERE scores. In the study cohort, the SOFA score demonstrates superior predictive accuracy compared with the GWTG-HF and ADHERE scores [the AUCs when predicting 30-day mortality are 0.769 (95% CI = 0.676–0.862) and 0.789 (95% CI = 0.705–0.873)]. Furthermore, the predictive accuracy of the SOFA score was assessed according to the LVEF. The AUC values for LVEF ≤ 20%, 20–30%, 30–40%, and >40% are 0.975 (95% CI = 0.911–1.000, *P* < 0.01), 0.864 (95% CI = 0.749–0.978, *P* < 0.01), 0.875 (95% CI = 0.729–1.000, *P* < 0.05), and 0.937 (95% CI = 0.822–1.000, *P* < 0.001), respectively.

### Kaplan–Meier analysis

**Figure 2.**
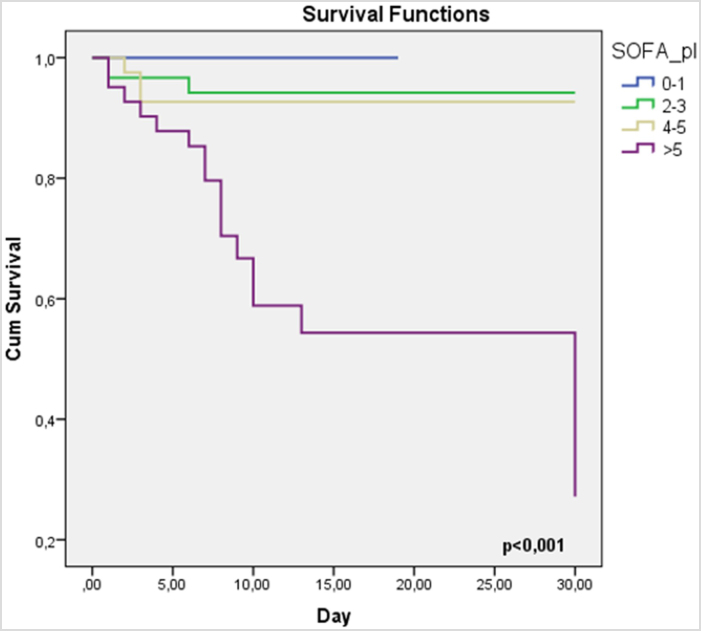
Kaplan–Meier analysis according to SOFA score. SOFA, sequential organ failure assessment.

We divided the patients into four categories on the basis of the SOFA score (SOFA score <2, 2–3, 4–5, and >5). Kaplan–Meier analysis shows that the increase in SOFA score is associated with an increase in mortality rate in the 30-day follow-up (*P* < 0.001) (Fig. 2). The survival rate significantly decreased when SOFA score was >5, and the majority of the event occurred within 15 days of admission. The cumulative survival was excellent when the SOFA score was <2.

### Predicting re-hospitalization

The performance of the SOFA score in predicting hospital readmission is poor, with an AUC of only 0.497 (95% CI = 0.352–0.641). When analyzing other scores, such as the ADHERE and GWTG-HF scores, these also showed limited ability to predict readmission rates, with AUC values of 0.592 (95% CI = 0.451–0.733) and 0.631 (95% CI = 0.499–0.763), respectively.

## Discussion

To our knowledge, this is the first study that evaluated the prognostic value of the SOFA score in hospitalized patients with acute heart failure with complete data on LVEF. Additionally, a significant number of patients (40.2%) in the study have low LVEF (<30%). In our study population, the SOFA score (calculated at admission) shows a significant correlation with in-hospital mortality and 30-day mortality. Higher SOFA scores are associated with lower survival rates, particularly within the first 15 days of hospitalization. The SOFA score also demonstrates better predictive value than the GWTG-HF and ADHERE scores for both in-hospital mortality and 30-day mortality. However, the SOFA score is not able to predict hospital readmission.

Efforts have been made to determine a score that can assess the risk of death in patients with ADHF. The use of tools to predict mortality in patients with ADHF has two main purposes. The first is the avoidance of unnecessary hospitalization in patients who have a low risk of death (short term, such as within 7 days or 30 days). Some authors believe that the proportion of patients with acute heart failure in the emergency department who can be treated as outpatients is up to 50%[10]. The Emergency Heart Failure Mortality Risk Grade for 7- and 30-day mortality scores are risk scores that help determine the mortality rate in patients with ADHF[11]. In the very-low or low-risk group, the 7- and 30-day mortality rates are both 0%, thus showing that outpatient treatment can be safely performed in these subjects.^[10-11]^ The second is the stratification of the risk of mortality in patients hospitalized for ADHF, particularly those in cardiovascular ICUs (CICUs). The identification of higher-risk subjects helps clinicians better individualize treatment regimens and promotes the use of invasive/adjuvant therapies instead of treating all patients by using the same approach[12].

In our study, as well as in other studies using the SOFA score as a prognostic tool,^[4,5]^ the primary purpose is to stratify inpatients. Patients in the current study have an average SOFA score of 3.14 ± 2.67, with SOFA scores >2 accounting for 65.7%. Although SOFA was initially used to predict the prognosis of patients in septic shock, there is increasing evidence of its prognostic value in patients in CICUs[2]. Given that 65.7% of patients have SOFA scores >2, the overall mortality rate in the study population is expected to be high. In fact, up to 72% of patients in the current study were treated in the CICU, with the overall mortality rates in groups with SOFA scores >2 and <2 being 0% and 15.5%, respectively. In our study, the C-reactive protein levels measured at admission did not show substantial differences across the SOFA score subgroups. Nonetheless, infectious events, such as pneumonia, are recognized as significant contributors to mortality in patients with ADHF.

Many risk scores have been developed to assess risk in patients with ADHF. Studies with large sample sizes have used the following scales: Enhanced Feedback For Effective Cardiac Treatment-Heart Failure (EFFECT-HF), Barthel Index-Enhanced Feedback for Effective Cardiac Treatment (BI-EFFECT), Frailty plus BI-EFFECT (FBI-EFFECT), GWTG-HF, Brigham and Women’s Hospital, and ADHERE[13]. When evaluating stratification capability, FBI-EFFECT has a c-statistic value of 0.76; this value is significantly higher than that of EFFECT-HF and BI-EFFECT, which have c-statistic values of 0.64 and 0.72, respectively[14]. However, to determine the prognostic value of the FBI-EFFECT score, it is required to combine information from the different variables of the three component scores, including the Fried modified criteria, Barthel Index, and Heart Failure Risk Scoring System-Enhanced Feedback for Effective Cardiac Treatment risk score,^[14,15]^ thereby greatly limiting its applicability in clinical practice. Risk scores such as GWTG-HF[7] and ADHERE[6] are simpler and externally validated with greater practicality; however, their limitations include the following: First, they lack the completeness present in multi-organ assessment (ADHERE score only uses two parameters: BUN and SBP)[6]. The pathogenesis of acute heart failure not only involves the circulatory system but also includes other organ systems with the cardiovascular system at the center; every deterioration in hemodynamics and cardiac output affects other organs and vice versa. Second is the excessive use of fixed parameters such as age, race, and history of chronic obstructive pulmonary disease (GWTG-HF scale)[7]. It is clear that the clinical parameters of patients with acute heart failure vary from hour to hour and day to day of treatment; therefore, the use of dynamic parameters will improve the assessment accuracy for the conditions of these subjects[4].

The SOFA score has been evaluated in a number of other studies, with the following basic conclusion: a higher SOFA score is correlated with a higher in-hospital mortality rate and 30-day mortality regardless of age, gender, or other comorbidities[4]. In general, the SOFA score shows good prognostic value in the populations of these studies, with AUC > 0.7,^[2,4,5]^ and is equivalent to the prognostic ability of SOFA in patients with sepsis, which is 0.753[16]. Jentzer studied 9961 patients and obtained the highest predictive value of the SOFA score, with an AUC value of 0.83 (95% CI = 0.813–0.843) for in-hospital mortality. However, the subjects of this study included patients with varied clinical conditions and not only focused on subjects with cardiovascular disease, particularly patients with acute heart failure; therefore, the SOFA score of these patients might be high without accompanying heart failure^[2,4]^. In the current study, the AUC values for in-hospital mortality and 30-day mortality are 0.895 (95% CI = 0.825–0.965) and 0.897 (95% CI = 0.830–0.964), respectively (Table 2); these values show the excellent mortality prediction ability of the SOFA score. LVEF should also be considered a factor in predicting mortality because a lower LVEF is associated with increased mortality. However, the predictive ability of the SOFA score is maintained when assessed at different ranges of ejection fraction (LVEF values of <20%, 20–30%, and >30%).

**Table 2:** Predictive accuracy of different risk scores

Risk scores	In-hospital mortality	*P* value	30 days Mortality	*P* value
	AUC (95% CI)		AUC (95%CI)	
SOFA	0.895 (0.825–0.965)	<0.001	0.897 (0.830–0.964)	<0.001
ADHERE	0.788 (0.702–0.875)	<0.001	0.789 (0.705–0.873)	<0.001
GWTG-HF	0.781 (0.687–0.874)	<0.001	0.769 (0.676–0.862)	<0.001

ADHERE, Acute Decompensated Heart Failure National Registry; AUC, area under the curve; GWTG-HF, Get With the Guidelines-Heart Failure; SOFA, sequential organ failure assessment.

It is also necessary to identify some factors that may lead to an overestimation of the prognostic ability of the SOFA score in the study population. The mortality rate in our study is 10.2%, mainly in the group with SOFA score >4 (mortality in this group accounts for 86.3% of the total). The group with SOFA score >5 accounts for only 19% of the study population but includes 72.5% of non-survivors. This may incorrectly increase the prognostic value of SOFA. Although there is an imbalance in the original data, when using the GWTH-HF or ADHERE score, the prognostic value of these scores is lower than the SOFA score (in-hospital mortality: AUC values of 0.781 and 0.769 for GWTH-HF and ADHERE, respectively; 30-day mortality: AUC values of 0.788 and 0.789 for GWTH-HF and ADHERE, respectively). Another point that needs attention is that the proportion of patients with LVEF <30% is mainly distributed in the group with SOFA scores ranging from 0 to 1, which is the group with in-hospital and 30-day mortality rates of 0. This shows that the cardiovascular component is not the main factor leading to patient death but that multi-organ disorders combined together are important factors that contribute to the increased mortality rate in subjects with ADHF.

Our study also has some limitations. First, this is a single-center study with a small sample size. Given the limited sample size, the study may have lacked sufficient power to identify an association between the SOFA score and 30-day readmission rates. Second, although this study was performed in a specialized cardiovascular center, patients hospitalized for ADHF may have secondary conditions such as pneumonia or acute renal failure, which can lead to increased SOFA values where heart failure is only a contributing factor and not the main cause of hospitalization. Third, the SOFA value is only calculated on the first day of hospitalization; therefore, it is not possible to evaluate the prognostic ability of the SOFA score at different periods during the treatment process in relation to the patient’s mortality rate.

The 2021 Surviving Sepsis Campaign guidelines advise against relying solely on qSOFA and SOFA as screening tools for sepsis, in favor of other early warning scoring systems[3]. This recommendation is based on concerns regarding the sensitivity and specificity of these tools when used independently. This consideration is crucial when applying these scoring tools for other purposes.

Although the SOFA score demonstrated potential in predicting outcomes within our cohort, it should not be utilized in isolation. Instead, it should be incorporated into a comprehensive clinical assessment. Future research should focus on validating these results and investigating how SOFA can be integrated with other risk assessment tools.

## Conclusion

Patients with acute heart failure who have high SOFA scores have higher short-term mortality. The SOFA score can be used as a complementary tool when identifying patients who are at high risk of death, and this approach can assist clinicians in planning monitoring and treatment. Future studies are needed to confirm the results of this study.

## Data Availability

Datasets generated during and/or analyzed during the current study are publicly available, available upon reasonable request.

## Research registration unique identifying number (UIN)

This research has been registered in Clinical Trials. UIN: NCT06354426. https://www.clinicaltrials.gov/study/NCT06354426.
